# A decade of outsourcing in health and social care in England: What was it meant to achieve?

**DOI:** 10.1111/spol.13036

**Published:** 2024-05-21

**Authors:** Anders Bach‐Mortensen, Benjamin Goodair, Christine Corlet Walker

**Affiliations:** ^1^ Department of Social Policy and Intervention University of Oxford Oxford UK; ^2^ Department of Social Sciences and Business Roskilde University Roskilde Denmark; ^3^ Centre for the Understanding of Sustainable Prosperity University of Surrey Guildford UK

**Keywords:** adult social care, children's social care, health care, marketisation, outsourcing

## Abstract

The increased private provision of publicly funded health and social care over the last 75 years has been one of the most contentious topics in UK public policy. In the last decades, health and social care policies in England have consistently promoted the outsourcing of public services to private for‐profit and non‐profit companies with the assumption that private sector involvement will reduce costs and improve service quality and access. However, it is not clear why outsourcing often fails to improve quality of care, and which of the underlying assumptions behind marketising care are not supported by research. This article provides an analysis of key policy and regulatory documents preceding or accompanying outsourcing policies in England (e.g., policy document relating to the 2012 and 2022 Health and Social Care Acts and the 2014 Care Act), and peer‐reviewed research on the impact of outsourcing within the NHS, adult's social care, and children's social care. We find that more regulation and market oversight appear to be associated with less poor outcomes and slower growth of for‐profit provision. However, evidence on the NHS suggests that marketisation does not seem to achieve the intended objectives of outsourcing, even when accompanied with heavy regulation and oversight. Our analysis suggests that there is little evidence to show that the profit motive can be successfully tamed by public commissioners. This article concludes with how policymakers should address, or readdress, the underlying assumptions behind the outsourcing of care services.

## 1 INTRODUCTION

The increased private provision of publicly funded health and social care services over the last 75 years has been one of the most contentious topics in UK public policy (Children England, 2016; Corlet Walker et al., 2022; Krachler & Greer, 2015). In the last decades, health and social care policies in England have consistently—directly and indirectly—promoted the outsourcing of public services to private third sector and for‐profit companies with the assumption that private sector involvement will reduce costs and improve service quality and access (Le Grand, 1991). Outsourcing is intended to improve outcomes through market mechanisms, such as increased competition among providers and user choice.

Outsourcing has largely been implemented by procuring health and social care services in open tender, and by shifting more commissioning and market management responsibilities towards local authorities and other public sector agencies. To this end, recent UK legislation of health and social care has been designed to promote and enforce private sector involvement. In consequence, there has been a significant expansion of the private (both for‐profit and non‐profit) sector delivery of health and social care services.

Table 1 shows a summary of the current outsourcing uptake and provision changes in the last 10 years. In short, the table demonstrates that adult and children's social care services are increasingly outsourced, and that private sector delivery of National Health Service (NHS) is also on the rise. There are multiple ways to measure outsourcing, and we are limited by the data made available by public agencies in the three sectors. While we report the outsourced activity (e.g., NHS treatments, weeks of adult residential care, and children's social care placements), we could also have reported the levels of expenditure, which provide very similar trends (see DFE, 2022; NHS Digital, 2023; Rahal & Mohan, 2022).

**TABLE 1:** Overview of sectors.

Sector	Regulator	Commissioner	Outsourcing uptake	Percentage change in last decade (2011–2022)
National Health Services	Care quality commission	Clinical commissioning groups	9.1% private sector in 2022* 90.9% public	3.4% private in 2011 (6.5%‐point increase) 96.6% public (6.5%‐point decrease)
Adult social care	Care quality commission	Local authorities	96.5% private sector in 2022** 3.5% public	88% private in 2011 (8%‐point increase) 12% public (8%‐point decrease)
Children's social care	Ofsted	Local authorities	38% for‐profit in 2022*** 7% third sector 47% public	29% for‐profit in 2011 (9%‐point increase) 2% third sector (5%‐point increase) 62% public (15%‐point decrease)

* Percentage of completed inpatient or outpatient treatments by NHS or private providers—treatments exclude non‐consultant led discretionary services.

** Percentage of weeks residential care provided to 65+ group in ‘own provision’ or ‘external provision’.

*** Percentage of children in care placed with for‐profit, third sector or LA providers. The percentages do not add up to 100 due to family‐based provision, which is not displayed in this table.

*Source*: NHS: RTT waiting time data (NHS England, 2023b); Adult social care: Adult Social Care Activity and Finance Report (NHS Digital, 2023); Children's social care: SSDA903 returns (DFE, 2022), data and calculations to be made available upon publication.

This development has prompted a growing focus on the unintended consequences, for example in terms of the increased instability in those parts of the market, and how marketisation in some cases has led to a reduced supply of services. There is a growing body of academic work that connects outsourcing to poorer user and service outcomes (Bach‐Mortensen, Goodair, & Barlow, 2022; Bach‐Mortensen, Murray, et al., 2022; Goodair & Reeves, 2022; Patwardhan et al., 2022). At the same time, recent policy documents report that the data on the changing provision landscape often fails to meet the needs of users, and that the effectiveness of outcomes is not always known by government departments (House of Lords Economic Affairs Committee, 2019; Housing Communities and Local Government Committee, 2019; National Audit Office, 2021). This suggests that the impact of pro‐private market reforms has not been well documented.

## 2 OBJECTIVES

The primary focus of this study is on the policy material related to outsourcing within the NHS, adult social care (e.g., nursing homes), and children's social care (e.g., children's homes). Specifically, we aim to investigate how policy documents justified the outsourcing of healthcare and social care and whether these justifications vary across different sectors. While the academic rationale for outsourcing is clear in terms of improving outcomes and reducing costs via market mechanisms, a growing scholarship is questioning whether the market can reliably improve health and social care service, due to, among other things, the complexity of such provision and the risk of misaligning financial and care‐related values (Corcoran & Albertson, 2023; Corlet Walker et al., 2022; Goodair & Reeves, 2024a). To expand on the empirical work on market failure in health and social care, this article will review and identify the specific intentions of outsourcing policy and implementation as reported in relevant legislation and policy documents.

Previous studies in this area have focused on the politics of outsourcing in relation to the long‐term debates and underlying preferences leading to outsourcing (Grimshaw, 2013; Pollock et al., 2002), or on the experience of outsourcing from the commissioner perspective (Bach‐Mortensen, Goodair, & Barlow, 2022; Bach‐Mortensen, Murray, et al., 2022; Goodair, 2023; Needham et al., 2022). This previous work allows us to consider the narratives and debates and think about the discourses perpetuated by political actors. However, understanding the concrete voiced policy and delivery intentions behind outsourcing is critical for evaluating and implementing such reforms effectively, and for holding the government to account for their intended outcomes.

This article seeks to achieve three objectives:

1. Clarify the intended mechanisms, as stated in relevant legislation and accompanying policy documents, through which outsourcing in health and social care services were meant to improve public services.
2. Assess whether the implementation of outsourcing aligns with the mechanisms intended to achieve those improvements.
3. Compare outsourcing lessons between the NHS, adult social care, and children's social care.

To achieve these objectives, we will begin by introducing the concept of outsourcing and outline the mechanisms through which it is expected to improve public services. We will then analyse policy and regulatory documents that preceded or accompanied outsourcing policies, such as the policy document related to the 2012 Health and Social Care Act and the 2014 Care Act. Last, we discuss shared lessons based on our analysis of each sector.

## 3 APPROACH AND METHODS

The article is structured as follows. We will begin by introducing the concept of outsourcing and outline the mechanisms through which it can improve or degrade public services. This initial step provides the backdrop for our subsequent analysis. Following this introduction, we will identify and review key policy and regulatory documents that have advocated for the outsourcing of public services, covering outsourcing legislation and policy materials pertaining to the NHS, adult social care, and children's social care.

This article aims to identify the reported mechanisms and intended effects that marketisation and outsourcing were designed to achieve within the domains of health and social care. It is these voiced intentions, which make the empirical contribution of this research. We employ an inductive approach to defining outcomes and quality of care as reported by official documents. We will then focus on two specific reforms in England: The 2012 Health and Social Care Act for the NHS, and the Care Act, 2014 for adult social care. We will also analyse outsourcing material related to children's social care, for which there is an absence of specific reform.

It is important to note that our analysis is centred on England, and we do not aim to delve into historical developments in outsourcing beyond the last decade. We also do not explore the political determinants of outsourcing, as this has been extensively covered elsewhere (Gingrich, 2011). As such, the focus is on recent trends in outsourcing within these sectors.

We have chosen to concentrate on health care, residential adult services, and children's social care for several reasons. First, these sectors have experienced a significant surge in outsourcing, especially in the last decade. Second, they have all experienced a sustained and significant increase in demand since 2010 (Baker, 2024; CQC, 2022b; DFE, 2022). Third, each sector operates under different commissioning structures and regulatory frameworks, making it valuable to examine the outsourcing structure and intentions separately. Finally, these sectors share meaningful similarities, as they all provide care to populations with the shared goal of safeguarding and enhancing the quality of life for service users.

## 4 WHAT IS OUTSOURCING IN THE CONTEXT OF HEALTH AND SOCIAL CARE?

Outsourcing is a term used to describe the process of contracting public services to the private sector (Sasse et al., 2019). This is distinct from privatisation, which describes the process of assets transferring ‘from public to private hands’ (Jensen & Stonecash, 2005, p. 769). The outsourcing of health and social care services have some unifying features: services are publicly funded via taxation, services are organised in markets with regional commissioning bodies given the responsibility of procuring services on behalf of the public, and services are provided by a range of private and third sector organisations under the regulation and monitoring of an independent regulator.

Outsourcing practices and implementation varies across the health and social care sectors. In the NHS, outsourcing of secondary care typically refers to the performance of routine operations as patients can sometimes choose to receive, for instance, NHS‐funded cataracts, hip, knee operations from a private sector provider. The unique aspect of NHS secondary services is that most services are provided in large public hospitals and therefore the NHS also outsources many of its hospital management, facilities management, and IT services. In adult social care, outsourcing describes the purchasing of beds from a predominantly private market of residential and nursing homes for aging residents. It can also include services for at‐home‐care and for working‐age adults. The unique aspect of adult social care is that there are a lot of self‐funded residents who self‐fund their services. Finally, children's social care outsources services to provide therapeutic and residential care (e.g., children's homes), for children who are either taken into the care of the local authority and away from their previous family setting—or those identified as at risk of requiring this. The unique context of children's social care is the provision of services often without the input of the service‐user, and sometimes without their consent.

### 4.1 Outsourcing health and social care: How the market replaces the state

Outsourcing is a practice that has gained considerable traction in public service delivery (Petersen et al., 2018). At its core, outsourcing involves contracting out specific functions or tasks of public services to private companies, including for‐profit and third sector (non‐profit) organisations. The ideal process for achieving this in theory is through fair competition in markets between service providers who are incentivised to cater and adapt to the needs and preferences of service users. There are typically two separate allocation mechanisms. The first is the commissioning of services by a public body (often regional commissioner, local government, or health board)—who is in control of the public finances, arranges contractual conditions for provision, and regulates who is eligible to provide health or social care services. The second allocation mechanism is the decision‐making power granted to service‐users, who are often given some agency in deciding who they receive care from. These two separate mechanisms are both intended to build in competition and accountability into the provision of public services by the private market and ultimately to benefit the needs and preferences of public commissioners and service‐users.

The theorised benefits of outsourcing in the public sector encompass various dimensions, including cost efficiency, increased flexibility, and the potential for accessing specialised expertise. By entrusting specific services to private providers, governments and public institutions aim to focus their resources on core functions, thus potentially reducing operational costs. Further, outsourcing can provide the flexibility needed to adapt to changing demands and circumstances, ensuring that public services remain responsive and efficient (Weisbrod, 1989). However, it is well known and documented that social care and outsourcing markets differ from conventional markets on a number of key dimensions (Gash et al., 2013; Knapp et al., 2001). Most importantly, service outcomes are often too complex and poorly defined to measure and monitor reliably, meaning that commissioners are vulnerable to opportunistic provider incentives. Even if outcomes can be measured, contracting out complex services is costly due to the transaction costs involved in monitoring and managing such contracts (Brown et al., 2015; Petersen et al., 2018). Consequently, the expectations of better outcomes and cheaper services are often not easily realised in practice.

Next, we present our analysis of the policy intentions of outsourcing for the NHS, children's social care, and adult social care, respectively.

## 5 NATIONAL HEALTH SERVICE: INCREMENTAL PRIVATISATION, RADICAL REFORM

The 2010s saw incremental and creeping increases in healthcare privatisation in England. This shift in the provision and priorities of the NHS was enabled by significant reforms. The NHS differs from adult and children's social care provision in England in that the public ownership of hospitals is a central design feature of the service. The NHS was founded through a widespread nationalisation of hospitals, creating a service delivered by the state both in terms of funding and provision. Consequently, the recent increase in outsourcing of services is a fundamental departure from the historical intentions for the NHS. One legacy of the nationalised history of the NHS is that a large proportion of healthcare is still delivered by publicly owned providers.

The major reform since 2010 to the commissioning and outsourcing policies in the NHS was the 2012 Health and Social Care Act (HSCA). The policy reformed the organisations conducting healthcare commissioning, the legal requirements placed upon the procurement processes, and the providers licensed to deliver NHS services. The 2012 act was a radical attempt to make competition the modus operandi and to expand the competition towards an external market of providers. This change led to criticisms that the passing of the HSCA represented ‘the final frontier’ (Pollock & Price, 2011, p. 294) and that the act was an attempt at ‘prise open the NHS oyster’ (Reynolds & McKee, 2012, p. 131).

Table 2 summarises the stated intentions of this development, and the policies that were meant to support their implementation. The 2012 HSCA provided the legal framework to (1) enforce competitive fair‐play in terms of avoiding anti private sector sentiments, (2) promote and incentivise private sector involvement, and (3) promote free choice among patients.

**TABLE 2:** Intended effects of NHS outsourcing.

Intentions	Elaboration	Interpretation
‘Protecting patients from anti‐competitive abuses against their interests’	A key aim of the 2012 Health and Social Care Act was to ‘enshrine a fair‐playing field’ between the range of providers competing to deliver services for the NHS—and to explicitly encourage to include providers ‘from charity or independent sector’ (Department of Health Factsheet A1, 2012, p. 1). The promotion of competition was sought via empowering independent for‐profit and non‐profit healthcare providers, who were deemed to have previously been experiencing ‘specific abuses and unjustifiable restrictions that demonstrably act against patients' interests’ (Department of Health Factsheet C4, 2012, p. 1). This intention was supplemented by the following policies: That the commissioning board and clinical commissioning groups may not ‘engage in anti‐competitive behaviour which is against the interests of people who use such services’ (HSCA, 2012, sec. 75) Outlawing interventions by the secretary of state, commissioning board or regulator to change the proportion of public or private providers (HSCA, 2012, secs. 13P, 62 and 147) Outlawing procurement decisions based on public or private ownership status (The National Health Service Regulations, 2013, sec. 3) Creating the independent regulator, monitor, with powers to investigate on its own initiative whether the commissioning board or a clinical commissioning group has failed to comply with laws banning anti‐competitive behaviour (HSCA, 2012, sec. 76) Give monitor powers to direct a relevant body to put in place measures for the purpose of preventing failures to comply with a requirement (HSCA, 2012, sec. 76)	Aimed to ensure private companies treated just the same as NHS trusts in commissioning Underlying argument that NHS trusts were preferred pre‐reform—and that this was bad for patients Aimed to achieve improvements by outlawing ‘anti‐competitive behaviours’
‘Liberating the provision of NHS services—increasing the quantity and variety of providers’	Alongside an effort to reduce any ownership bias in the commissioning process, the HSCA wanted to enable more providers to compete for and deliver NHS services. The 2011 white paper argues to ‘free up provision of healthcare, so that in most sectors of care, any willing provider can provide services’ (Department of Health, 2010, p. 37). This was justified in terms of increasing ‘innovation, improvements and productivity’ through intensified competition (ibid). The concrete legislated policies implemented to achieve this intention include: Instructing monitor to create a licensing system for independent providers and NHS foundation trusts, with powers to enforce the licensing requirements (HSCA, 2012, chap. 3) Outlawing the exclusion of qualified providers from patient choice options, framework agreements, or contract bidding (The National Health Service Regulations, 2013, sec. 7) Instructing the commissioning board to create frameworks for which commissioner pays for which services. In practice, this enabled private providers to deliver and be compensated for services without a contract with the commissioner (HSCA, 2012, sec. 147) Repealing the restrictions on private treatments from the 2006 Health and Care Act (HSCA, 2012, sec. 165)	Intended to expand who provided NHS services Underlying argument that innovation existed in new and different kinds of providers Aimed to achieve this via procurement and choice processes having to include all providers
‘Giving patients choice and control by increasing accountability and patient voice’	The patient choice agenda was an instrumental element of the NHS reforms furthered by the HSCA. The white paper expresses this intention as: ‘patients will be at the heart of everything we do. So they will have more choice and control, helped by easy access to the information they need about the best GPs and hospitals’. (Department of Health, 2010, p. 1). This interestingly excluded staff whistleblowing (Freedom to Speak Up, 2015), The concrete legislated policies implemented to achieve this intention include: Instructing commissioners to procure enough services to enable user choice (HSCA, 2012, sec. 14V) Duties to patient choice were conferred to the new commissioning board and commissioning bodies (HSCA, 2012, secs. 23 and 26) Requires an information standard to be published for commissioners and providers of health services for the NHS (HSCA, 2012, sec. 250). Created Healthwatch England and local Healthwatch whose statutory responsibilities are to involve patients in the commissioning, provision, and monitoring of healthcare (HSCA, 2012, part 5, chap. 1) Give the independent regulator, monitor, powers to impose requirements on commissioners to ensure they protect and promote the right of patients to make choices (HSCA, 2012, sec. 75) A requirement for alternative providers to be offered within the regulated conditions (The National Health Service Commissioning Board and Clinical Commissioning Groups (Responsibilities and Standing Rules) Regulations, 2012, regulation 48(4))	Intended to expand the choice patients have over who provides their care Underlying belief that it is best if patients can seek care bespoke to their preferences and needs Aimed to achieve this via requiring the implementation of patient choice of different stakeholders—including commissioners

The increased use of for‐profit providers was intended to deliver better quality of care for less cost. However, there is little evidence to suggest this was achieved. Rather, evidence has found that areas with highest increases in outsourcing and the most deprived areas have seen greater deterioration in the quality of care (Goodair & Reeves, 2022; Watkins et al., 2017). The failures of this policy direction resulted in a service with increasing levels of patient dissatisfaction, worsening health outcomes and a more burnt‐out workforce, even before the outbreak of COVID‐19 (Bimpong et al., 2020; Green et al., 2017; Honeyford et al., 2017).

There are several possible reasons for why the 2012 Act has not improved the quality of care, including misaligned incentives by for‐profit providers or knock‐on effects of outsourcing on public hospitals. However, it was the competitive aspect of provider selection that was the least popular within the NHS hierarchy, and by the end of the 2010s NHS England suggested a more collaborative approach to commissioning. Specifically, the 2019 long‐term plan suggested removing ‘the counterproductive effect that general competition rules and powers can have on the integration of NHS care’. (NHS England, 2019, p. 113). A similar sentiment was reported by the government 3 years later in the 2022 Health and Care Act: ‘We believe collaboration, rather than competition, as an organising principle, is a better way for the NHS and the wider health and care system to respond to today's challenges’ (DHSC, 2022).

Competition was thus replaced with collaboration in the 2022 HSCA, and the health sector regulator's duty to enforce fair play competition was removed. Perhaps more significantly, regulations requiring competitive tendering are to be removed. Commissioners will thereby be able to award tenders to a provider of their choice and can no longer be penalised for favouring public or non‐profit sector provision. However, the intention to ‘liberate the NHS’ and ‘open the NHS social market up’ has not been altered and integration is still defined through the shared provision of services between public and private providers, although only non‐profit sector providers are mentioned explicitly (Department of Health, 2010, p. 39; DHSC, 2022). Meanwhile the formal regulation over who can provide services remains the same. A new licensing scheme is being introduced in line with the policy changes, removing competition demands and inserting collaborative demands. Yet many of the updated requirements for licences, for example to support system working or achieve net zero carbon emissions, do not apply to private providers (NHS England, 2023a).

## 6 CHILDREN'S SOCIAL CARE OUTSOURCING: RAPID OUTSOURCING, LITTLE REGULATION

Of the three sectors explored in this article, children's social care has seen the largest changes in private provision since 2010, with children's homes having largely transitioned to the for‐profit sector. Historically, for‐profit private sector outsourcing has been restricted to placements for looked after children, such as children's homes and foster care. Since 2010, private sector delivery of these services has massively increased and is now the dominant provision type of residential and foster care. To‐date, less than 10% of all children's homes are operated by local authorities, which is a decrease of 23%‐points since 2010.^1^

Table 3 summarises the key intentions of outsourcing children's social care. An interesting way that the children's social care sector stands out from the adult and health care, is that the role of outsourcing is much vaguer in legislation. There have been no major legislative reforms to the procurement, licensing, or regulating of providers of children's social care placements since the marketised system was largely established in 1989. This absence in terms of intentional reform relating to outsourcing was highlighted in the 2022 Competition and Market Authority's (CMA) children's social care market study, which found that competition through open tender does not appear to be the result of ‘deliberate policy choices’:

‘[…]the placements market as it operates today is not the result of deliberate policy choices by national governments on how children's social care should be delivered, but rather a reaction by multiple local authorities, voluntary providers and private providers to a range of factors—including regulatory developments, financial constraints and reputational risk—that have played out over time’. (CMA, 2022, p. 36)

**TABLE 3:** Intended effects of children's social care outsourcing.

Intentions	Elaboration	Interpretation
Increasing the number and quality of placements through competition	In 2006 and 2007, the Blair government published the Care Matters green and white papers (DFE, 2006, 2007), which outline a comprehensive list of challenges in the sector and present an ambitious list of reforms on how to fix them. Among other suggestions, the articles report that competition is key to improving the quality and quantity of placements, and that the government wants to achieve ‘a good mix of local authority, private and voluntary sector provision’ (DFE, 2006; pp. 45–46). Similarly, the subsequent white paper Care Matters: Time for change highlights the importance of the private sector in improving outcomes for children in care: ‘The private sector has much to offer children in care’ (DFE, 2007, p. 13). For example, instead of restricting private provision, the Narey review on residential care urged more non‐profit providers to enter the market in order to ‘offer a competitive challenge to private sector providers’ (Narey, 2016, p. 18). Further, the 2016 ‘Putting children first’ DfE report pushed for more local authorities to deliver a ‘mixed economy of delivery models’ (DFE, 2016, pp. 44–45). Most recently, one of the three main recommendations by the CMA market study was to reduce barriers to market entry for new providers (CMA, 2022)	Intended to increase the supply and variation in the providers of children's social care Underlying argument is that expansion and enough places can be best achieved by the private sector
Introducing desirable incentives to social workers	New labour attempted to achieve intensified incentives for quality in social work services through a piloted scheme of independent ‘social work practices’. The idea involved social workers becoming independent contractors, either as third sector or for‐profit companies—similarly to general practitioners working for the NHS. The concept was explored by Le Grand's, 2007 report Consistent care matters, which examines the feasibility of outsourcing statutory social work duties to the private sector. The working group concluded that the programme offers great potential to improve service quality, and that ‘a profit sharing social work practice will encourage a new dynamic—one that rewards responsiveness, industry and effectiveness, while penalising indifference and inefficiency’ (Le Grand, 2007, p. 27). The implementation of social work practices was eventually evaluated by an independent group of researchers, who found that ‘None of the 10 commissioners or finance officers interviewed in the local authorities contracting with SWPs [social work practices] considered that the SWP model had resulted in savings for the local authority’ (Stanley et al., 2013, p. 33). The experiment was reported to at best be cost neutral, and, at worst, to have been more costly than local authority provision	Wanted to outsource the employment of social workers to the private sector Underlying argument is that social workers would react to fiscal responsibility by increasing their productivity Aimed to achieve this with rolling out an experiment—which was ultimately rejected after negative evaluation
Separate commissioning from service provision	The idea that local authorities should transition from providers to buyers of children's services was explicitly suggested in the 2016 report chaired by Le Grand on the ways forward for the (failing) Birmingham's children's service. This suggestion was further explored in a subsequent government commissioned report conducted by Laingbusson, which was tasked to ‘provide an analysis of the existing mixed market in the provision of children's social care services in England, and the potential for developing the capacity and diversity of provision in England’ (LaingBuisson, 2016, p. 25). The report encouraged scaling up private sector outsourcing for all statutory duties. It concluded that a privately run children's social care system is not just desirable, but also feasible: ‘[…]a competitive market could be established in most areas of the country, given a flow of tenders from commissioners’ (LaingBuisson, 2016, p. 18). Although the suggestion to outsource statutory children's services was rejected by the government, commissioning continues to be sold as a silver bullet for the sector. The main recommendations posed in the children's social care study by the CMA involve increasing the supply of private placements by improving the commissioning capacity and expertise of local authorities. Both the recent CMA (CMA, 2022) and Care Review (MacAlister, 2022) reports recommended that this could be achieved by restructuring local authority commissioning into pooled regional care cooperative units. This recommendation was accepted by the government and is due to be piloted in 2024	Wanted to limit local authority's primary role to purchaser of services (rather than provider) The underlying argument was that markets need a strict separation between purchaser and provider to allow level‐playing fields and selection based on quality of care Wanted to achieve this by expanding the use of the market into other statutory care (e.g., foster and adoption)

As shown in Table 3, private sector outsourcing was meant to achieve three objectives: (1) to help local authorities access more placements, (2) to improve quality by improving incentives among social workers and placement providers, and (3) to enable local authorities to customise their social care needs as the commissioner rather than the provider of placements. After several decades of private sector growth, the success of the implementation regarding the above objectives are unclear at best.

One thing is clear: children's social care is in disarray. Numerous reports describe how sector provision has deteriorated in recent years (Children's Commissioner, 2019, 2020a, 2020b; LGA, 2021). This is illustrated by the fact that the number of children in care has never been higher (DFE, 2022), and that many children are illegally accommodated in unregulated accommodation, more than 44% of all children are placed outside their local authority, and placement stability remains a large concern. These developments have occurred simultaneously with the growth of private sector provision, and recent research links negative outcomes with the growth of for‐profit provision (Bach‐Mortensen et al., 2023; Bach‐Mortensen, Goodair, & Barlow, 2022; Bach‐Mortensen, Murray, et al., 2022). This indicates that outsourcing has not achieved its intended aims of improving quality and access to placements.

What is also clear is that outsourcing and private sector commissioning are still being sold as the solution to the sector's problems. The main recommendation posed in both the CMA and MacAlister reports that was embraced by the DfE was to develop regional care cooperatives to improve the expertise, leverage, and bargaining power of children's social care commissioners. Furthermore, the DfE reports that this will improve LA negotiation leverage: ‘[…]our proposals on regional commissioning above will give regions greater buying power and put them in a stronger position when negotiating with private providers’ (DFE, 2023, p. 196). The rationale for this recommendation is intuitive: by pooling resources and expertise at regional level, the commissioning capacity and negotiation leverage will improve. Yet, the focus on commissioning, rather than service provision, rests on an assumption that the best path to improving outcomes for children is to align the for‐profit motive of private providers with the interests of children in care through commissioning.

This is a contestable assumption for three reasons. First, there is no national oversight or framework that enables contract monitoring or evaluation on child‐level outcomes. Fundamentally, outcomes are not clearly defined in the sector, and commissioners are over reliant on Ofsted ratings that are not designed to guide commissioning decisions (Bach‐Mortensen, Murray, et al., 2022). Second—and in contrast to adult social care—there is no market oversight scheme for children's commissioners (CMA, 2022), meaning that there are no national oversight or safeguards against poor commissioning practices at local authority level. Third, there is a critical lack of available data to assess service and commissioning performance. Even the heavily pro‐outsourcing Laingbuisson report acknowledges that there is not sufficient data to evaluate outsourcing performance: ‘The need for baseline data is paramount to enable the comparison of results before and after implementing significant reform. The absence of such makes it very difficult to determine if outsourcing has succeeded’. (LaingBuisson, 2016, p. 126).

There is a growing body of evidence that can be used to assess recent developments. First, it is well documented that the number of private sector placements has significantly increased in the last decade (Bach‐Mortensen, Goodair, & Barlow, 2022; Bach‐Mortensen, Murray, et al., 2022). Second, many private providers have achieved abnormally high profit margins from local authorities (CMA, 2022), and the CMA report found that the level of private sector profits did not reflect a well‐functioning market. Third, recent research has found that the quality—measured by Ofsted ratings—is worse among for‐profit children's homes compared with local authority provision (Bach‐Mortensen, Goodair, & Barlow, 2022; Bach‐Mortensen, Murray, et al., 2022). Last, there is a severe shortage of available places in the sector, which is so acute that Ofsted has declared it a ‘sufficiency crisis’ (Ofsted, 2021b, p. 1).

This recent evidence and research into outsourcing echoes what has been highlighted by sector stakeholders for decades: that for‐profit outsourcing does not enable local authorities to achieve better outcomes for children (ADCS, 2023; Children England, 2016; LGA, 2021). This is perhaps partly explained by the fact that it was never explicitly intended to operate as a market. As noted by Children England: ‘There is lack of effective strategic oversight in the market for residential care because it was never decisively designed to be a competitive market, and doesn't work at all well as one’ (Children England, 2016, p. 11). The conservative government continues to reject restricting for‐profit provision, claiming that doing so will worsen the scarcity of an already insufficiently supplied market (CMA, 2022; DFE, 2023). Both the Scottish and Welsh government have reached a different conclusion and are currently shifting towards a public and third sector delivery model.

## 7 ADULT SOCIAL CARE: THE END OF A PUBLIC SERVICE

Adult social care in England has undergone a progressive privatisation through outsourcing since the enactment of Margaret Thatcher's National Health Service and Community Care Act in 1990 (Knapp et al., 2001). This makes it the most well‐established case of outsourcing examined in this article, which is reflected in the proportion of private providers in the market. The act had the effect of changing the role of local authorities from being primarily responsible for delivering adult social care, to gradually becoming responsible for coordinating and commissioning it from the private market (Barron & West, 2017; The King's Fund, 2006). The act also embedded the concept of consumer choice, enabling individuals to choose which provider to receive care from. Additionally, there was a growing focus from the former Audit Commission on the value for money achieved through their commissioning practices (The Health Foundation, 2023). In the three decades following the Community Care Act 1990, the adult social care provisioning landscape changed dramatically. In the residential care sphere, it has transitioned from predominantly local authority provision in the 1980s, to 83% for‐profit providers, 13% voluntary organisations, and just 3% delivered directly by local authorities by 2019 (IPPR, 2019).

The shift towards marketisation that began in the 1990s was based on two important assumptions; namely that it would improve consumer choice and deliver more efficient care services. These assumptions have then been re‐embedded in more recent legislation, affirming the government's commitment to marketisation and the ideas behind it. One of the major policy reforms of note within the adult social care sector since 2010 was the Care Act, 2014, which explicitly stated that the responsibilities of Local Authorities are to promote the ‘efficient and effective operation of a market in services for meeting care and support needs’ (Care Act, 2014; part 1 sec. 5(1)).

These new market‐shaping responsibilities for local authorities laid out in the Care Act, 2014 were introduced with three core aims (DHSC Factsheets, 2016):

1. to ensure that people ‘receive services that prevent their care needs from becoming more serious, or delay the impact of their needs’ (DHSC, 2016; Factsheet 1)
2. to ensure that people ‘can get the information and advice they need to make good decisions about care and support’ (DHSC, 2016; Factsheet 1)
3. to ensure that people ‘have a range of provision of high quality, appropriate services to choose from’ (DHSC, 2016; Factsheet 1)

Table 4 summarises each of these aims. These pieces of guidance highlight the clear expectation that competition and consumer choice would drive more efficient and higher quality services in localised markets overseen by local authorities.

**TABLE 4:** Intended effects of adult social care outsourcing.

Intentions	Elaboration	Interpretation
Improve people's independence and wellbeing	The first stated aim behind the Care Act, 2014 was that local authorities should ensure that people have access to the services they need, when they need them, with the aim of preventing their care needs from escalating and supporting their independence and wellbeing. This intention was represented in the Care Act Part 1, sec. 2: ‘A local authority must provide or arrange for the provision of services, facilities or resources, or take other steps, which it considers will—(a) contribute towards preventing or delaying the development by adults in its area of needs for care and support; (b) contribute towards preventing or delaying the development by carers in its area of needs for support; (c) reduce the needs for care and support of adults in its area; and (d) reduce the needs for support of carers in its area’. The act was meant to prevent people from developing care and support needs by considering what ‘services, facilities, and resources are already available in the area (for example local voluntary and community groups), and how these might help local people’ (DHSC, 2016; Factsheet 1). This goal is primarily aimed at identifying and fostering private services within communities, coupled with early identification of people who might need access to those services, positioning the local authority as a facilitator rather than a provider of services	Intended to promote independence for care users Underlying belief that independence equates to independent from state‐provided care Aimed to achieve this by changing the state's role from provider to facilitator
Improving access to information to support choice	The second stated aim of the Care Act, 2014 was to improve access to information, to support consumer choice. This is enacted in legislation through part 1, sec. 4: ‘a local authority must establish and maintain a service for providing people in its area with information and advice relating to care and support for adults and support for carers’. This information service must provide information on the types and range of care and support services available, the processes for accessing them, where people can go for financial advice, routes for raising concerns about safety and wellbeing of people currently receiving care (factsheet 1). This focus of the act likely came partially in response to the findings of the 2011 caring for our future survey and subsequent white paper of the same name, which lists lack of access to good information as one of the key challenges for people accessing the care system at that time (HM Government, 2012)	Intended to improve information people can access on care services Underlying belief is that people can navigate a mixed‐market well once they have enough information Aimed to achieve this by requiring local authorities to provide this information
Increasing the diversity of high‐quality providers through commissioning	The third stated goal of the Care Act, 2014 was to ensure that a diversity of high‐quality providers was available to services users, and that this should be achieved by relying on market forces This intention is represented in the Care Act, 2014 part 1, sec. 5): ‘A local authority must promote the efficient and effective operation of a market in services for meeting care and support needs with a view to ensuring that any person in its area wishing to access services in the market—(a) has a variety of providers to choose from who (taken together) provide a variety of services; (b) has a variety of high quality services to choose from; and (c) has sufficient information to make an informed decision about how to meet the needs in question’. The government's market shaping guidance, which was provided to local authorities in 2017, named the core market shaping activities as: engaging with stakeholders to better understand supply and demand within the local area; signalling what types of services are needed ‘now and in the future’ to the market; to ‘encourage innovation, investment and continuous improvement’; and finally to empower individuals purchasing their own services to be ‘efficient consumers’ (DHSC, 2017). The local government association, the national membership body for local authorities in England and Wales, explained that such market shaping was expected to come about ‘primarily through commissioning quality, outcomes‐based services that focus on wellbeing’ (LGA, 2014, p. 10)	Intended to diversify adult social care provision Underlying belief that variation in service provider brings innovation and competition Aimed to achieve this by requiring local authorities to manage their local markets

The 2014 Care Act intended for outsourcing to improve the well‐being of adults in care, promote user choice, and increase the quality of services. In doing so, it intended to ‘[…]dissolve the traditional boundaries that lie between the third sector, private organisations, local authorities and individuals’ (HM Government, 2012, p. 3). In practice, private sector services have gradually continued to increase as public provision has effectively disappeared from the sector (CQC, 2023b).

Increased outsourcing has been accompanied with a decrease in residential care provision in recent years, with an 1.5% decline in the number of care home beds between 2012 and 2019 (Bayliss & Gideon, 2020); a trend that has continued over the course of the COVID‐19 pandemic (CQC, 2022a, 2022b), even though demand for care has increased as the population has continued to age (Dilnot, 2017). This shift has been acknowledged by the regulator, CQC, who not only noted in their 2022 report that ‘capacity in adult social care has reduced and unmet need has increased’, but also reflected on the impacts this was having on hospitals' ability to discharge patients, with only 2 in 5 who are ready to leave being able to do so (CQC, 2022a, 2022b, p. 4). This situation was exacerbated by the COVID‐19 pandemic, but these trends were occurring before 2020. For example, the charity Age UK found back in 2019 that there were 1.5 million older adults with some kind of unmet care need (Age UK, 2019).

The impact of improving information as a route to supporting consumer choice rests on two important assumptions. First, that access to ‘clear, comparable information’ will enable people to make choices in line with their preferences, which will in turn support their wellbeing. Second, that increasing access to information will ‘encourage providers to up their game’ (HM Government, 2012, p. 3). Both assumptions rely on the premise that consumers are able and/or willing to freely act on their preferences when selecting or changing providers. This is arguably a questionable assumption because there are very high switching costs associated with changing care providers. Moving between care homes has been linked to ‘increased anxiety, depression, and fall risk’ (Corlet Walker et al., 2022, p. e299). As such, using improved information to promote consumer choice may not be a realistic mechanism for motivating quality improvements among providers.

The assumption that competition is an effective mechanism through which to achieve high‐quality services has not been supported by the research. A 2014 analysis of 10,000 care homes found that ‘quality and price were reduced by greater competition’ (Forder & Allan, 2014, p. 73). A finding that has been echoed by analyses from the USA (Bowblis, 2012; Castle, 2005). This is likely for two reasons. First, providers have a limited ability to respond to competition by implementing labour saving technologies, for example due to the time‐intensive nature of care work (Corlet Walker et al., 2022). Second, local authorities have substantial market power since they purchase services on behalf of a large number of individuals, making up approximately half of the revenue that goes into the adult social care sector (Kotecha, 2019). This means that they can cap prices at lower than they would be in a competitive market (Corlet Walker et al., 2022). Indeed, as a result of austerity motivated welfare policies, prices have been unsustainably low (DHSC, 2023), and the 2016 CMA report that current fee levels do not cover the operating costs of care (CMA, 2018). Hence, competition among private providers can end up driving prices down to unsustainably low levels, and research is already revealing that self‐funded residents are subsidising the costs of state‐funded residents (Henwood et al., 2022).

The establishment of the market oversight scheme as part of the Care Act was intended to offset risks associated with a profit‐oriented care market (CQC, 2023a). However, the scheme did not give the CQC the power to intervene with providers at risk of failing. The 2022 HSCA was accompanied with more regulatory powers including a clause giving the Secretary of State the power to require care providers to give more detailed provider information, and the expansion of the market oversight role of the CQC (CQC, 2022a). The emphasis on enhanced information and oversight are meant to be gearing local authorities towards improved emergency response, market oversight, and care planning. Like in children's social care, this suggests that private sector commissioning remains the main strategy employed by the central government to address the challenges in the sector.

## 8 DISCUSSION: DIFFERENT SECTORS, SHARED LESSONS?

This article has reviewed the legislation and policy intentions supporting the outsourcing within the NHS, children's, and adult social care. Table 5 summarises our findings, which are unpacked in the three lessons below. Table 5 includes a discussion of implementation outcomes.

**TABLE 5:** Market oversight and outcomes in health and social care.

	NHS	Adult social care	Children's social care
For‐profit growth in the last decade	Low	High	High
Market oversight	Strong: The NHS licensing scheme sets requirements for providers; regulators are given powers to enforce competition	Weak: The CQC market oversight scheme assesses difficult to replace providers and shares information with LAs but comes with no power to act on the information	Very weak: No market oversight. Ofsted cannot regulate poorly performing provider chains (Ofsted, 2021a)
User agency	Strong: In theory, patient choice is strongly protected in legislation. Choice and agency have historically been higher for affluent populations (Beckert & Kelly, 2021)	Variable: Good patient choice in theory, but research shows that many adult social care residents do not act as regular consumers—they are ‘sticky’ (Corlet Walker et al., 2022, p. e299), and there are both financial and personal costs to changing provision (Knapp et al., 2001)	Very weak: The nature of children's social care poses significant challenges in achieving meaningful user choice. A 2022 analysis of local authority sufficiency strategies found that children's voices are not considered in the provision planning (Bach‐Mortensen, Murray, et al., 2022)
Implementation outcomes	Poor in places: A growing body of evidence suggests system level harms of outsourcing (Goodair & Reeves, 2022; Toffolutti et al., 2017)	Poor: Unmet need increasing (CQC, 2022a, 2022b); competition pushing quality down (Forder & Allan, 2014); council and non‐profit sector provision outperforms private provision (Bach‐Mortensen et al., 2024; Barron & West, 2017; Patwardhan et al., 2022)	Severely poor: More children are placed out of their home area (DFE, 2022), sufficiency crisis (Ofsted, 2021a), reports of abuse, unregulated accommodation on the rise (Foster, 2021), and lower quality private provision (Bach‐Mortensen, Goodair, & Barlow, 2022)

### 8.1 First lesson: Poor market oversight appears to be correlated with accelerated for‐profit sector growth

Each sector explored in this article is subject to a different level of market oversight, with regulation being relatively strong in the NHS, and weak for adult and children's social care. Our review of the developments in the last decade shows that for‐profit growth has been significantly higher in sectors with less regulation. NHS outsourcing following the 2012 HSCA was, however, accompanied with significant market oversight, and the establishment of a licensing scheme and several independent patient interest groups.

Conversely, the marketisation of adult social care has been accompanied with much less oversight, even though outsourcing has been an integral part of its provision since 1990 (Knapp et al., 2001). The only oversight mechanism is the market oversight scheme, which involves the CQC identifying and informing local authorities about providers in their area that would be difficult to replace if they were to close (CQC, 2023a). In theory, this enables local authorities to plan for provider failure and to shape their provision in a way that considers the risk of provider closure. However, the scheme only involves risk assessment and information sharing, and the CQC does not have the power to intervene in or prevent provider failure.

Not only is there no market oversight scheme in children's social care, but Ofsted does not have a mandate to regulate underlying providers of children's services (Ofsted, 2021a). This applies to adult social care too, in that the CQC can only regulate individual provider settings and cannot penalise systematic poor performance by underlying provider chains.

### 8.2 Second lesson: The evidence suggests that commissioners’ ability to control the profit‐motive is poor

A common trend underlying the outsourcing development in all three sectors is the push for public agencies to assume the role of commissioner rather than service providers. Incentives serve as both the selling point and the chief concern in terms of commissioning private companies to deliver health and adult social care services. If competition does not incentivise the pursuit of ‘good’ outcomes, the quality of care may decline, and users may be adversely affected. The question is then: how can you trust a company to prioritise users and patients over costs and the bottom‐line? The answer by proponents of outsourcing is simple: by incentivising the delivery of meaningful outcomes through contracting and monitoring. The selling point of private sector involvement work is thus that it enables commissioners to activate desirable incentives among private providers through competition on relevant outcomes. This requires commissioners to engage in market stewardship, typically defined as the ‘the long‐term oversight of market mechanisms, as well as the commissioning process’ (Gash et al., 2013, p. 20).

Numerous reports, including two recent CMA investigations, have found that adult and children's social care commissioners are not able to effectively shape the private market (Bach‐Mortensen, Murray, et al., 2022; CMA, 2018, 2022; CQC, 2022a, 2022b), even though private provision represents the majority of these services. This is evidenced by the fact that sufficiency and unmet need remains a severe problem in both sectors as private provision has increased, even though this is the main area private provision was meant to improve. This is an important finding, because localised market stewardship is conventionally considered the most effective commissioning model (Dickinson et al., 2022), but the evidence suggests that decentralised commissioning has not operated well under the condition of low supply in terms of available number of places in adult and children's social care.

This challenge is exacerbated by the absence of data on outsourcing performance. In fact, the data on the provider market of both adult and children's social care is critically poor (Curry & Oung, 2021). As a consequence, commissioners do not have the necessary data and information to develop long‐term market planning. Moreover, it presents a challenge in terms of promoting learning in the sector, in that the absence of data on commissioning outcomes makes it difficult to research the impact of alternative commissioning initiatives, as well as risk factors of poor market outcomes. There is also a critical absence of evidence around the impact of different contract types, even though this is the key link between provider and commissioners (Jensen & Stonecash, 2005). The lack of evidence has continuously been identified as a barrier to market reform. Yet, it does not seem to have impacted government support in the commissioning of these services.

In the NHS, concerns with profit motives are often focused on the cherry picking of patients. This behaviour has been evidenced in a range of acute care services over many years in the NHS (Cooper et al., 2018; Mason, 2012; Owusu‐Frimpong et al., 2010). As a consequence, public hospitals end up serving higher proportions of difficult‐to‐treat patients. The evidence about the prevalence of cost‐cutting by private healthcare companies is scarce, but there is research showing that for‐profit hospitals treating more profitable patients, cutting their staffing levels, and resulting in worse health outcomes (Goodair & Reeves, 2024b).

### 8.3 Third lesson: Private sector commissioning has become entrenched in health and social care

Social policies and the institutional norms that shaped them are known to be path dependent. It is difficult to undo and rewind the effects of inherited legislation (Pierson, 2000). It is therefore not surprising that the norms and ideologies that shaped a given policy continue to influence subsequent reform, even if the wording and circumstances change. We find that the underlying principles of outsourcing remain entrenched in health and social care policy, even in initiatives that discursively depart from its original intent.

For children's social care, the announcements of the regional care cooperatives shows that the English government continues to push for a delivery model that assumes that private sector commissioning is the best strategy to maximise value for public money. The same can be observed in adult social care. The 2022 HSCA puts an even greater emphasis on the market shaping duties of local authorities than the 2014 Care Act, and thus further embeds and reinforces the role of local authorities as commissioners of private sector provision.

In regard to the NHS, the 2022 HSCA marks a discursive departure from competition to collaboration (a shift that was not made in adult social care), competition through patient choice continues to be encouraged. The potential for the direct awarding of services and the integration of care to result in less detrimental forms of outsourcing remains uncertain. However, the concerns raised over the past decade provide ample cause for alarm.

## 9 LIMITATIONS

This article relies on evidence produced since these reforms to assess the processes that have been observed in each sector over the last decade. However, this article does not systematically review the empirical evaluations in each sector—something that is a rich vein for further research. This article evaluates the voiced intentions from official policy documents. This means that we do not seek to understand political intentions not laid out in official material. One such example may be to reduce the cost of provision. This decade represented a period of state retrenchment through long‐term austerity programmes in England. It may well have been the case that the underlying aim of outsourcing was to cut service costs (see e.g., the analysis of (Corcoran & Albertson, 2023)), but it is not the ambition of this article to assess the underlying politics of outsourcing. Throughout the analysis, we have focused broadly on outsourcing to private providers (i.e., both third and for‐profit sector providers), and have not discussed how these policies affected these sectors differently. This is an important area for future research.

Finally, we do not know how the outcomes in the last decade in these sectors compared to alternative scenarios in which the services were provided primarily by the state or non‐profit independent providers. This does not negate the poor outcomes seen in the outsourced portion of the market; we can confidently say that outsourcing does not appear to have solved the problems in these sectors as intended. However, it means that we cannot at this point tease apart the relative impact of, for example, austerity versus the impacts of outsourcing itself.

## 10 CONCLUSION

This article has identified and analysed the intentions of outsourcing. Going forward, more empirical work is needed to diagnose the extent to which outsourcing is the cause or symptom of the sufficiency crisis, and how this development coincides with other drivers, such as the austerity motivated cuts (Glasby et al., 2020). Both children's and adult social care funding has been critically low for decades, and past work has found this to be a driver of poor quality. Further work should unpack the interaction between outsourcing and the broader policy context. It is also important to note that commissioning and market shaping can take different forms (Needham et al., 2022). Future research should investigate how different approaches to commissioning are associated with different market outcomes.

Despite the persistent promotion of outsourcing as a solution to improve outcomes and save costs in health and social care, there are still ongoing problems with unmet need, poor quality outcomes and insufficient supply, indicating that outsourcing has not achieved the results it was intended to. Our review of the academic research and policy literature reveal multiple patterns in the impact of outsourcing across the three care contexts. We find that more regulation and market oversight appear to be associated with less poor outcomes and slower growth of for‐profit provision. However, evidence on the NHS suggests that marketisation does not seem to have achieved the intended effects of outsourcing, even when accompanied with regulation and oversight. In line with previous research and theory, our analysis suggests that there is little evidence to suggest that the profit motive can be tamed by public commissioners. This evidence appears to be ignored by recent policy, in that commissioning and competition continue to be sold as a silver bullet to improve outcomes and save costs, especially in adult and children's social care.

## Data Availability

Data sharing is not applicable to this article as no new data were created or analyzed in this study.

## ENDNOTE

^1^
Calculations according to registration dates of current (September 2023) children's homes.
